# Control of an outbreak of invasive Group A *Streptococcus* in a care home in Lincolnshire, England

**DOI:** 10.1017/S0950268825100204

**Published:** 2025-06-24

**Authors:** Natalie Davison, Jennifer Clements, Victoria Pruteanu, Joanne Enstone, Theresa Lamagni, Kartyk Moganeradj, Yan Ryan, John MairJenkins, Kerry Roulston, Bethan Stoddart, Jharna Kumbang

**Affiliations:** 1 East Midlands Health Protection Team, UK Health Security Agency, Nottingham, UK; 2 Health Protection Team, NHS Lincolnshire Integrated Care Board, Lincoln, UK; 3 Nursing and Quality Directorate, Lincolnshire County Council, Lincoln, UK; 4 Public Health Microbiology Division, UK Health Security Agency, London UK; 5 Antimicrobial Resistance & Healthcare-Associated Infection Division, UK Health Security Agency, London, UK; 6 Field Services Midlands, UK Health Security Agency, Nottingham, UK; 7 Path Links Microbiology, United Lincolnshire Hospitals Trust (ULHT), Lincoln, UK

**Keywords:** care home, iGAS, infectious disease, outbreak, public health

## Abstract

In March 2024, the East Midlands Health Protection Team was notified of a case of invasive Group A *Streptococcus* (iGAS) infection in an elderly care home resident. Twenty-two days later, another case in a resident from the same floor of the care home was notified. In accordance with national guidelines, an outbreak was declared, and a multidisciplinary outbreak control team (OCT) was urgently convened. Screening for GAS throat carriage was undertaken for staff and residents, excluding those receiving end-of-life care. All isolates were strain typed and characterised. Infection prevention and control (IPC) visits were undertaken to provide ongoing support. Screening identified five residents and five staff members positive for GAS. Antibiotic prophylaxis was provided to all staff throughout the setting (*n* = 74) and all residents on the affected floor (*n* = 35). Three individuals were positive on repeat screening. All staff and residents screened negative after 4 months and the two clinical cases recovered. Eleven of the 12 GAS isolates were identified as *emm* 3.93. This outbreak highlighted the importance of rapid screening, possible only through the deployment of a dedicated team, and rescreening post-decolonising treatment, as a means to contain such outbreaks.

## Background

*Streptococcus pyogenes* (Group A *Streptococcus*, GAS) can cause a range of diseases, from non-invasive manifestations such as pharyngitis and impetigo to more severe, life-threatening invasive disease (iGAS), such as bacteraemia, necrotising fasciitis, or streptococcal toxic shock syndrome [1]. GAS is highly contagious and can be transmitted through respiratory particles and direct contact with infected people or contaminated surfaces and objects [2]. Care homes are places where people live in later life to receive extra support with personal care, such as eating, washing, dressing, and taking medication. Elderly individuals (aged 75 years and older) and care home residents are at higher risk of GAS bacteraemia than the general population, and at higher risk of more severe outcomes, including death [3, 4]. In England, the rate of iGAS notifications in the 75 years and older age group (8.8 per 100,000) was considerably higher in the current 2023/2024 season compared to the rate observed in the five previous seasons (2017/2018 to 2021/22) which have a range of 1.5 to 3.9 [5]. In this outbreak report, we describe a GAS outbreak in a care home in Lincolnshire, England caused by *emm* sequence type 3.93 and the control measures used to contain it.

## Outbreak detection

In March 2024, the East Midlands Health Protection Team in the United Kingdom Health Security Agency (UKHSA) was notified of a case of iGAS infection in an elderly care home resident (Case A) who was admitted to hospital with cellulitis to the left hand. GAS was isolated from blood cultures taken on admission. Initial risk assessment of residents and staff in the care home was performed following UKHSA guidelines [6]. The home housed 74 residents, over two floors. The ground floor had 29 residents and five in bungalows. The first floor facilitated dementia care (35 beds) and contained a small end-of-life (EOL) unit, comprised of five beds. There were 74 members of staff, most of whom worked across the whole home. No symptomatic residents or staff were identified; however, a household contact of a staff member was clinically diagnosed and treated for GAS several weeks previously.

As the index case had a single room, there were no household-type contacts identified who met the criteria for considering chemoprophylaxis. A ‘Warn and Inform’ letter was shared with the manager of the care home for dissemination, with advice to be vigilant regarding other potential cases among residents and staff. Just over 3 weeks later (day 23), the UKHSA received notification of a further case of iGAS infection in a resident from the same care home on the same floor (floor 1, dementia unit). The second case (Case B) was admitted to the hospital 21 days after the notification of Case A. Case B was treated for pneumonia and systemic sepsis. GAS was isolated from blood cultures taken on admission, indicating acquisition in the community.

As two iGAS cases had been notified from the same care home setting in a short time interval, an outbreak was declared in accordance with national guidelines [6] and an outbreak control team (OCT) was urgently convened on day 24. A Consultant in Communicable Disease Control (CCDC) from UKHSA acted as its chair, with the care home manager, a clinical microbiologist, infection prevention and control (IPC) team, regional HPT team, Field Services, national epidemiology leads for streptococcal infection, Lincolnshire Integrated Care Board (ICB) and Local Authority (LA) in attendance.

## Method

### Case definition

Case definitions were agreed upon by the OCT as follows:

- A confirmed case was defined as any staff member or resident with an epidemiological link to the care home and laboratory confirmation of GAS *emm* type 3.93 between 23/03/2024 and 12/09/2024. Laboratory confirmation refers to the isolation of GAS by culture, in keeping with national guidance [5]. An iGAS case was defined as isolation of GAS from a normally sterile body site or GAS isolated from a non-sterile site in combination with severe clinical presentation, for example, streptococcal toxic shock syndrome (STSS), necrotising fasciitis.
- A probable case was any symptomatic case, for example, sore throat, skin/soft tissue infection between 23/03/2024 and 12/09/2024, with epidemiological links to a confirmed case linked to the care home from the date of notification of Case A.

### Case detection

Following the notification of Case B, the advice to remain vigilant for signs and symptoms of GAS infection in all members of staff and residents was reiterated to the care home. Based on subsequent reports of several staff with symptoms of a sore throat, with regular movement of staff between floors, a decision was made by the OCT to screen all care home residents and staff members, excluding the five End of Life residents residing on the first floor.

Following a collaborative OCT decision, the ICB arranged for the screening of the care home staff and residents. The ICB mobilised their Rapid Response Team (RRT) to attend the home from day 28 and undertook screening via throat swabs. The RRT consists of registered nurses and non-registered support workers trained in delivering vaccines, taking swabs, and other clinical assessments and interventions. The team is contracted to provide a health response under the direction of the ICB, both in and out of working hours.

### Microbiological investigations

Blood cultures were obtained on admission from the two hospitalised cases and processed by Path Links NHS Pathology. Screening swabs from care home residents and staff were also processed at Path Links NHS Pathology. Swabs were inoculated on blood agar and incubated for 24–48 hours. Isolates were identified by routine methods including latex agglutination, and confirmation and susceptibility testing performed by VITEK 2 system (bioMérieux, Lyon, France) Gram-positive identification (ID-GP). and Streptococcus Susceptibility cards.

All GAS isolates were referred to the UKHSA national Staphylococcus and Streptococcus Reference Service (SSRS) for *emm* gene typing by whole genome sequencing, using Illumina’s short-read Nextera XT library prep technology. The sequences derived were *emm* typed using the *emm* gene typing tool and characterised further at the single-nucleotide polymorphism (SNP) level using UKHSA’s PHEnix pipeline as described in Vieira et al. [7, 8].

### Site visit

A site visit was undertaken by a member of the Local Authority’s HPT team to assess iGAS outbreak control measures, cleaning and disinfection, and the management of on-site facilities. Areas of good practices were identified in the use of personal protective equipment (PPE), cleaning and disinfection, and hand hygiene. Actions were required related to the decontamination of equipment, and further training was recommended on aseptic technique and the management of wound care. The care home was advised to continue to deep clean affected areas, for example, floor 1, staff shared areas and rooms of confirmed cases, and ensure enhanced IPC measures remained in place. In response to these findings, a Senior Health Protection Nurse from the LA HPT delivered a bespoke IPC training session to care home staff working in the setting, and regular meetings were held with the care home management throughout the outbreak to provide additional support.

## Results

### iGAS case overview

**Figure 1.**
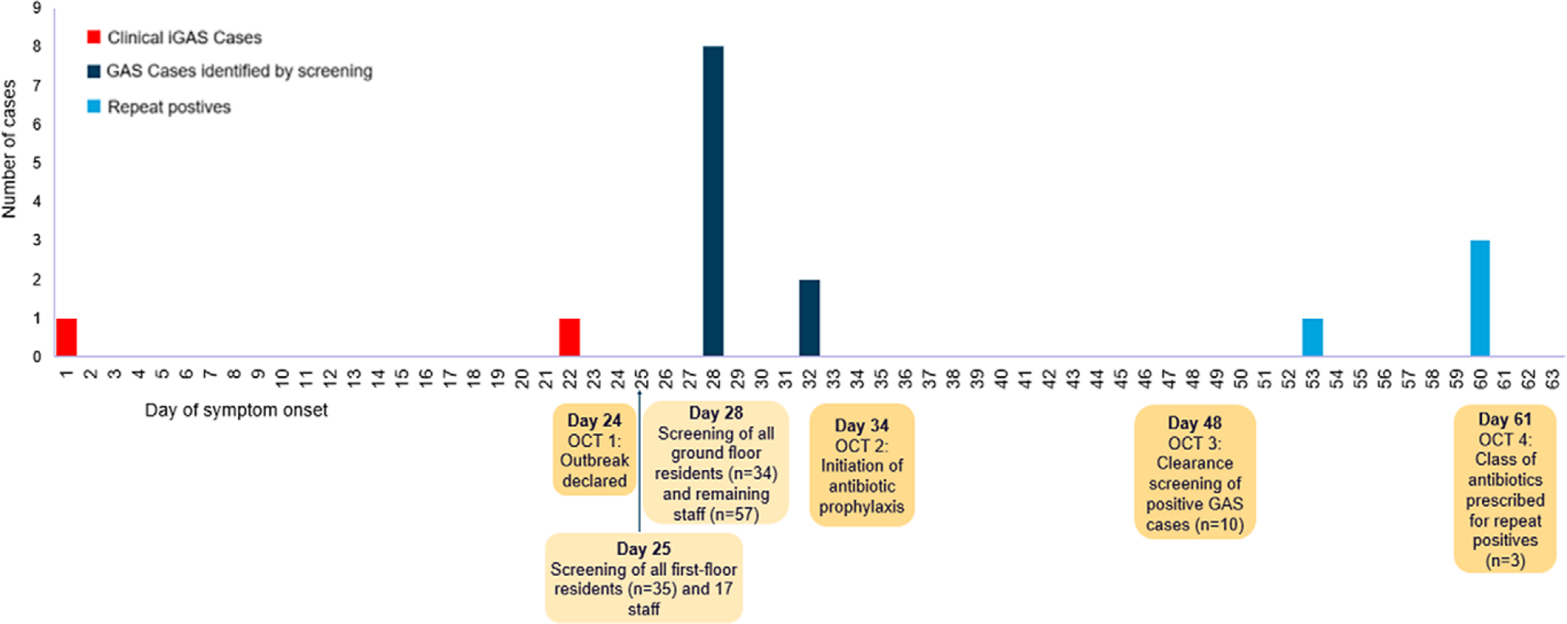
Distribution according to timing of symptom onset of confirmed invasive Group A *Streptococcus* cases (*n* = 2) and Group A *Streptococcus* cases identified from screening samples (*n* = 10) linked to an outbreak in a care home, Lincolnshire.

In total, there were two confirmed cases of iGAS identified during this outbreak, summarised in Table 1. Both cases resided on the first floor, dementia unit of the care home and made use of the dining and communal areas in the care home. A timeline of events is outlined in Figure 1. Both cases have recovered, with one case discharged back from the hospital to the care home and the other discharged to an alternative care home.

**Table 1.** Summary of confirmed invasive Group A *Streptococcus* cases linked to an outbreak in a care home, Lincolnshire, 2024 (*n* = 2 cases)

Case	Age category in years	Symptom onset	Day of blood cultures	Day notified	Floor of residence	Clinical presentation
A	>75	Day 0	Day 2	Day 2	Floor 1	Cellulitis to the left hand
B	>75	Day 21	Day 23	Day 23	Floor 1	Bronchopneumonia and systemic sepsis

### GAS screening results

Screening for throat carriage was offered to all staff members (*n* = 74) and all residents, excluding those receiving end-of-life care (*n* = 69). On day 25, first-floor residents (*n* = 35) and 17 staff members were screened via throat swabs. On day 28, all ground floor residents (*n* = 34) and 57 staff members were screened. A total of 10 cases were identified from throat swabs, including five staff members and five first-floor residents. No positive results were observed in ground-floor residents (Table 2).

**Table 2.** Outcome of screening for iGAS outbreak in a care home, Lincolnshire, 2024

Type of individual	Total no. individuals in home	Total no. individuals identified	No. GAS positives	%
Residents	74	69	5	7.2
Staff	74	74	5	6.7
All	148	143	10	7.0

### Microbiology results

**Figure 2.**
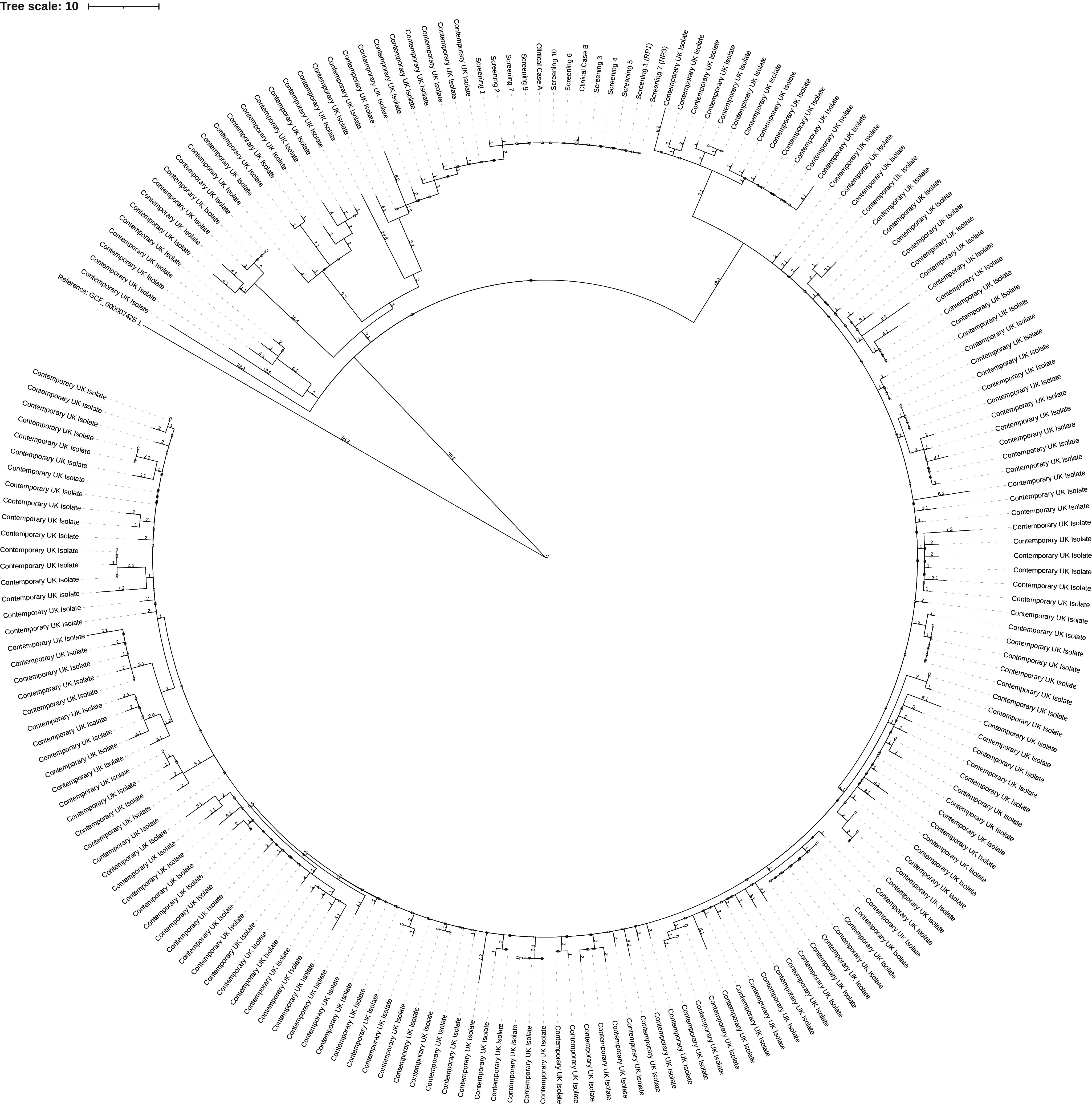
Phylogenetic tree of *emm* 3.93 outbreak isolates. The tree is made up of 202 different *Streptococcus pyogenes* emm 3.93 isolates, 189 contemporary, 13 from the investigation (two iGAS isolates, nine isolates via screening, two isolates via clearance screening). The investigation isolates are two SNPs from the nearest contemporary isolates. 567 variant positions that were non-recombinatoric as identified by Gubbins (Version 2.3.4) were used in its construction [8]. The figure was generated via iTOIL [9].

Both iGAS isolates, nine isolates identified from screening samples and two isolates from clearance screening were confirmed as *emm* type 3.93 (see Figure 2). A single cluster was identified through WGS analysis, as this demonstrated that all *emm* type 3.93 isolates fell within a 0–2 SNP cluster with nine isolates being genetically identical (see Table 3). A further case from a staff member was identified as *emm* type 4.0. For outbreak isolate accessions and associated metadata, see the supplementary table. Both blood stream isolates were fully sensitive to all antibiotics tested: Teicoplanin, Vancomycin, Clindamycin, Clarithromycin, Erythromycin, Penicillin, and Tetracycline.

**Table 3.** SNP matrix of *Streptococcus pyogenes emm* 3.93 isolates from an outbreak in a care home, Lincolnshire

	1530900	1491872	1468011	1495126	1495125	1491856	1488051	1523890	1491852	1491871	1491870	1491853	1491855
1530900	0	0	1	1	1	1	2	2	1	1	1	1	2
1491872	0	0	0	0	0	0	1	1	0	0	0	0	1
1468011	1	0	0	0	0	0	1	1	0	0	0	0	1
1495126	1	0	0	0	0	0	1	1	0	0	0	0	1
1495125	1	0	0	0	0	0	1	1	0	0	0	0	1
1491856	1	0	0	0	0	0	1	1	0	0	0	0	1
1488051	2	1	1	1	1	1	0	2	1	1	1	1	2
1523890	2	1	1	1	1	1	2	0	1	1	1	1	2
1491852	1	0	0	0	0	0	1	1	0	0	0	0	1
1491871	1	0	0	0	0	0	1	1	0	0	0	0	1
1491870	1	0	0	0	0	0	1	1	0	0	0	0	1
1491853	1	0	0	0	0	0	1	1	0	0	0	0	1
1491855	2	1	1	1	1	1	2	2	1	1	1	1	0

*Note*: The sequences were characterised at the single nucleotide polymorphism (SNP) level using UKHSA’s PHEnix pipeline [7]. The genetic relatedness is evident in this matrix as all emm type 3.93 isolates fell within a 0–2 SNP cluster with nine isolates being genetically identical.

### Outbreak control measures

Throughout the duration of the outbreak, four OCT meetings were convened to discuss the outbreak control measures. Following the identification of cases, the care home management and staff received IPC support, including a site visit with recommendations for enhanced cleaning, particularly in communal staff areas, and reinforcing the message regarding PPE, the use of face masks for staff during the outbreak, and the importance of good hand hygiene. A letter was circulated to staff and residents, which included ‘warn and inform’ information, with a low threshold for informing the Local Authority HPT of any further symptomatic staff or residents. The care home was advised to close admissions to reduce the risk of transmission to vulnerable population groups. A reactive media statement was produced in case any media queries were received. To ensure continued surveillance, the transfer of patient information between the care home and UKHSA was arranged via secure electronic accounts.

In response to the high proportion of GAS-positive screening results from first-floor residents (5/35), a decision was made to provide chemoprophylaxis to all residents on this floor. Isolation was also recommended for positive residents; however, it was recognised that this may be difficult due to their dementia diagnosis. Chemoprophylaxis was not provided to residents of the ground floor, as no positive results were observed in ground floor residents. Based on clinical assessment, the five End of Life residents residing on the first floor were also not prescribed chemoprophylaxis. All 74 care home staff members were offered chemoprophylaxis due to their exposure, as many worked across both floors of the care home and social mixing took place inside and out of work. Staff members positive for GAS were instructed to remain off work until 24 hours after commencement of treatment and safety netting advice given (specific guidance on what to do if the condition changes or fails to improve) if any other staff members started to feel unwell.

As per the national guidance, the antibiotic of choice for chemoprophylaxis was penicillin or clindamycin in the event of a penicillin allergy. The 10 GAS cases identified by screening received phenoxymethylpenicillin (500 mg every 6 h for 10 days). Following the administration of chemoprophylaxis and in accordance with the national acute guidance [10], repeat swabbing was undertaken at intervals of 24 hours and 1, 3, 6, and 12 weeks post completion of treatment. As shown in Table 4, at ‘week 1 post-treatment’ a positive GAS result (*emm* 3.93) was reported for a member of care home staff following a previous negative result at the first follow-up swab post-treatment (24 h). At ‘week 3 post-treatment’ repeat positive results for GAS were reported for two staff members (*emm* 3.93 and *emm* 4.0) and one resident (*emm* 3.93).

**Table 4.** Individuals identified with repeat positive clearance screens during iGAS outbreak in a care home, Lincolnshire, 2024

Resident/staff	*emm* type	Antibiotics prescribed (dose and duration)	24 Hours clearance screen	Week 1 clearance screen	Antibiotics prescribed (dose and duration)	Week 3 clearance screen	Antibiotics prescribed (dose and duration)	Week 1 restart clearance screen	Week 3 clearance screen	Week 6 clearance screen	Week 12 clearance screen	Catch up clearance screen
Resident (screening case 1)	3.93	Penicillin V^a^ (500 mg every 6 h for 10 days)	Negative	Negative	N/A	Positive (*emm* 3.93)	Penicillin V^a^ (500 mg every 6 h for 10 days)^a^/Rifampicin (300 mg twice daily for the last 4 days)	Negative	Negative	Negative	Negative	N/A
Staff (screening case 7)	3.93	Penicillin V^a^ (500 mg every 6 h for 10 days)	Negative	Positive (*emm* 3.93)	Penicillin V^a^ (500 mg every 6 h for 10 days)	Positive (Not submitted for *emm* typing)	Clindamycin (300 mg every 6 h for 10 days)	Negative	Negative	Negative	Not taken	Negative
Staff	4.0	Penicillin V^a^ (500 mg every 6 h for 10 days)	Negative	Negative	N/A	Positive (*emm* 4.0)	Clindamycin (300 mg every 6 h for 10 days)	Negative	Negative	Negative	Negative	N/A

a Phenoxymethylpenicillin (Penicillin V).

Following the three GAS repeat positives (two staff, one resident) at week 3 post-treatment swabbing, LA HPT continued to provide IPC support to the care home, with the first floor remaining closed to new admissions. However, as there were no resident cases on the ground floor, it was agreed that the care home could take ground floor admissions, with the assurance that staff remain separate for the two floors. All new admissions to the care home had a risk assessment completed, which was supported by the Local Authority HPT. At day 160, the final screening sample for the staff member who completed three courses of antibiotics was negative for GAS. The Consultant in Communicable Disease Control (CCDC) declared the outbreak over, but notifications of new cases were to be monitored as part of routine surveillance practices.

## Discussion

While transmission of iGAS is prevalent in the community [11, 12], outbreaks do arise, particularly in institutional care settings [13]. There is an increased risk in care homes where elderly residents with multiple co-morbidities are in close contact [14, 15]. Following the identification of this *emm* 3.93 iGAS outbreak in a care home facility, a rapid response by a multi-agency team appears to have brought this outbreak under control.

There is a diverse range of *emm* gene sequence types of GAS, with over 200 documented globally [16]. Analysis of reference laboratory iGAS isolate submissions from UKHSA for the 2023/24 season has showed that *emm* 3.93 was the most common gene type, overtaking emm 1.0 (the dominant type from last season) [5, 17]. Rapid turnaround on *emm* sequencing results confirmed a microbiological link between cases, in addition to the epidemiological link, which guided subsequent outbreak control measures. We recognised that there is evidence indicating excess risk of death for specific *emm*3 types [18], which required additional vigilance, given the vulnerable population involved in the outbreak.

In care home settings, the interval of iGAS cases may extend over several months and so no finite time limit to define an outbreak has been set, according to national guidance [6]. This can make the management of such outbreaks ambiguous and create variation in responses nationally. A systematic review by Barth et al. [19] highlighted the risks of nosocomial spread of GAS infections in aged care facilities, stating that these are increased where there are lapses in infection control measures. The delivery of IPC advice to the care home by the OCT and at the site visit is likely to have played an important role in the control of this outbreak as outlined in previous prolonged outbreaks in nursing homes [20].

Following an assessment of exposures, staff were hypothesised as the most likely transmission route, as staff movement was common between floors. As there is limited evidence on the most effective control measure for iGAS outbreaks in a care home [14], screening of staff and residents was decided as a first approach, rather than immediate chemoprophylaxis for all close contacts. This was to identify routes of transmission that could inform the public health response and minimise the exposure to antibiotics in the context of growing concerns about antimicrobial resistance and the risk of precipitating *Clostridioides difficile* infection (CDI). Based on the results of the screening, a subsequent decision was made to offer chemoprophylaxis to all first-floor residents and all staff members.

The UK guidelines for the management of contacts of iGAS infection in community settings [6] state that staff who were previously positive should be re-swabbed to check for clearance, with intervals as referenced from the acute health guidance [10]. In this outbreak, three of the 10 cases identified by screening failed microbiological eradication after a first course of antibiotics, with one case requiring three courses of antibiotics to achieve clearance. These repeat positive results signify the importance of maintaining the re-screening schedule to ensure eradication of carriage is successful. This need is further exemplified by a meta-analysis from McGuire et al. [21] which showed 9.1% (95% CI: 7.3–11.3) throat swabs collected after completion of antibiotic therapy were GAS culture-positive. We recognised the swabbing process was capacity intensive and required delivery at pace, which was only possible through the ICB’s RRT. There were challenges in adherence to the recommended repeat swabbing intervals due to the operational complexities of undertaking multiple re-swabs on multiple individuals. Further thought is required to understand the optimum time intervals for re-swabbing in community settings due to the extended time it takes to undertake testing and receive results. We suggest that other UKHSA HPTs may want to ensure that they have a robust process to address such an outbreak in their own regions.

In the general population, penicillin V results in the eradication of GAS in around 80% of cases [22] and is the recommended antibiotic for chemoprophylaxis in adults and children with no history of allergy [5]. In this outbreak, all GAS isolates were susceptible to all antibiotics tested, in keeping with national data that show antimicrobial resistance rates are <2% for tetracycline, erythromycin, and clindamycin in *emm* 3.93 iGAS isolates [5]. Two cases of *emm* 3.93 and one case of *emm* 4.0 remained positive following a 10 day course of penicillin V. One repeat positive case failed a second course of penicillin V, and eradication was eventually achieved following a course of clindamycin. The other repeat positive cases were prescribed clindamycin (staff), as per the national guidance, and penicillin/rifampicin (resident) to eradicate throat carriage. Due to their advanced age, the resident was felt to be at increased risk of CDI, so an alternative to clindamycin was offered [23]. Prescribing decisions are informed by a multitude of factors (e.g., site of colonisation, tolerability, and risk of side effects), but these findings highlight the importance of following national guidance [6] and prescribing a different class of antibiotics if penicillin fails to eradicate carriage. It is not known whether the repeat positive results were due to antimicrobial failure, or whether there was a medication adherence issue or re-acquisition occurred. Detailed genomic and biological investigations should continue to investigate this emergence.

Management of this outbreak was facilitated by strong inclusive partnerships present across the health system. Early engagement between health organisations allowed appropriate sharing of information and expertise to generate a collective understanding of the outbreak investigation which initiated swift public health action. Ongoing support throughout the investigation contributed to the effective multi-agency response, as well as promoting system working. Time between swabbing and results were reduced due to the utilisation of the RRT, and staff and residents at the home could commence prophylaxis sooner reducing the impact on the residents, staff and operational running of the care home.

## Supporting information

10.1017/S0950268825100204.sm001 Davison et al. supplementary material Davison et al. supplementary material

## Data Availability

The core genome nucleotide sequences of the isolates associated with this outbreak have been deposited in European Nucleotide Archive database. Accession number: PRJEB85543.

## Supplementary material

The supplementary material for this article can be found at http://doi.org/10.1017/S0950268825100204.
