# Supplementary material for: Control of an outbreak of invasive Group A Streptococcus in a care home in Lincolnshire, England
Source: Epidemiol Infect. 2025 Jun 24;153:e81. doi: 10.1017/S0950268825100204 (PMC12281235; doi:10.1017/S0950268825100204)
Supplement: Davison et al. supplementary material [file S0950268825100204sup001.docx]

**Supplementary Table: *S. pyogenes* *emm* 3.93 isolates from an outbreak in a care home, Lincolnshire**

| **Accession** | **Title** | **Sample Date** | **Isolation Site** | **Source** | **Staff/Resident** |
| --- | --- | --- | --- | --- | --- |
| ERS23137772 | 1468011 | 22/03/2024 | Blood culture | Clinical Case A | Resident |
| ERS23137773 | 1488051 | 12/04/2024 | Blood culture | Clinical Case B | Resident |
| ERS23137774 | 1491852 | 16/04/2024 | Throat swab | Screening 6 | Staff |
| ERS23137775 | 1491853 | 17/04/2024 | Throat swab | Screening 9 | Staff |
| ERS23137776 | 1491855 | 17/04/2024 | Throat swab | Screening 2 | Resident |
| ERS23137777 | 1491856 | 17/04/2024 | Throat swab | Screening 3 | Staff |
| ERS23137779 | 1491870 | 17/04/2024 | Throat swab | Screening 5 | Resident |
| ERS23137780 | 1491871 | 19/04/2024 | Throat swab | Screening 10 | Staff |
| ERS23137781 | 1491872 | 19/04/2024 | Throat swab | Screening 7 | Staff |
| ERS23137782 | 1495125 | 17/04/2024 | Throat swab | Screening 4 | Resident |
| ERS23137554 | 1495126 | 16/04/2024 | Throat swab | Screening 1 | Resident |
| ERS24508372 | 1523890 | 20/05/2024 | Throat swab | Screening 1 (RP1) | Resident |
| ERS24934815 | 1530900 | 13/05/2024 | Throat swab | Screening 7 (RP3) | Staff |
